# Facial Emotion Recognition Predicts Alexithymia Using Machine Learning

**DOI:** 10.1155/2021/2053795

**Published:** 2021-09-28

**Authors:** Nima Farhoumandi, Sadegh Mollaey, Soomaayeh Heysieattalab, Mostafa Zarean, Reza Eyvazpour

**Affiliations:** ^1^Department of Psychology, Faculty of Education and Psychology, University of Tabriz, Tabriz, Iran; ^2^Department of Cognitive Neuroscience, Faculty of Education and Psychology, University of Tabriz, Tabriz, Iran; ^3^Department of Biomedical Engineering, School of Electrical Engineering, Iran University of Science and Technology (IUST), Tehran, Iran

## Abstract

**Objective:**

Alexithymia, as a fundamental notion in the diagnosis of psychiatric disorders, is characterized by deficits in emotional processing and, consequently, difficulties in emotion recognition. Traditional tools for assessing alexithymia, which include interviews and self-report measures, have led to inconsistent results due to some limitations as insufficient insight. Therefore, the purpose of the present study was to propose a new screening tool that utilizes machine learning models based on the scores of facial emotion recognition task.

**Method:**

In a cross-sectional study, 55 students of the University of Tabriz were selected based on the inclusion and exclusion criteria and their scores in the Toronto Alexithymia Scale (TAS-20). Then, they completed the somatization subscale of Symptom Checklist-90 Revised (SCL-90-R), Beck Anxiety Inventory (BAI) and Beck Depression Inventory-II (BDI-II), and the facial emotion recognition (FER) task. Afterwards, support vector machine (SVM) and feedforward neural network (FNN) classifiers were implemented using K-fold cross validation to predict alexithymia, and the model performance was assessed with the area under the curve (AUC), accuracy, sensitivity, specificity, and F1-measure.

**Results:**

The models yielded an accuracy range of 72.7–81.8% after feature selection and optimization. Our results suggested that ML models were able to accurately distinguish alexithymia and determine the most informative items for predicting alexithymia.

**Conclusion:**

Our results show that machine learning models using FER task, SCL-90-R, BDI-II, and BAI could successfully diagnose alexithymia and also represent the most influential factors of predicting it and can be used as a clinical instrument to help clinicians in diagnosis process and earlier detection of the disorder.

## 1. Introduction

Alexithymia could be briefly described as emotional blindness [1] and refers to the difficulty in expressing and recognizing emotional states [2]. Alexithymic individuals misinterpret the somatic symptoms of emotional arousal, try to express their emotional distress through physical complaints, and seek treatment for their physical symptoms [3]. It is also argued that alexithymic individuals exhibit difficulties in understanding and regulating their emotions [4]. The prevalence of alexithymia in the general population is 10%–13%, and alexithymia symptoms are more prevalent among males [5].

Alexithymia, and particularly the subscale of “difficulty in identifying feelings,” is associated with various psychiatric disorders [6–10]. For instance, follow-up studies show that chronic alexithymia is consistently associated with depression and various symptoms of psychological disorders such as cluster-c personality disorders [11–13]. Moreover, the presence of alexithymia predicts poorer treatment outcomes for anxiety and somatoform disorders [14], depression [15], alcoholism [16], and mixed psychiatric disorders [17]. Alexithymia also seems to be a common feature of neurological diseases, with most evidence available for patients with traumatic brain injury, stroke, and epilepsy [18]. Therefore, identification of alexithymia may have important preventive, diagnostic, and therapeutic implications.

Clinical judgment is the most common approach to assessing alexithymia, which is questionable in terms of psychometric quality and its unknown interrater reliability [19]. The next method involves the use of questionnaires, such as the Beth Israel Hospital Questionnaire (BIQ) and the Toronto Alexithymia Scale (TAS-20), which is inexpensive and can be administered more rapidly [20]. Using only self-report tools and administering interviews have some limitations because the restricted introspection of subjects affects the results of the questionnaire [21], and interviews are more laborious to administer [20].

Over the recent years, clinicians have shown great interest in using analytical methods for efficiently diagnosing people with mental disorders from healthy individuals based on their scores. One of the methods used for this purpose is supervised machine learning (ML), which could automatically extract information from available data through creating different algorithms and techniques [22]. ML approaches are used in different areas, such as neuroimaging [22], malingering [23, 24], genetics [25], clinical medicine [26], and augmenting psychological tests [27]. The benefits of ML models in solving classification problems have been further proven [22]. Supervised ML methods allow for characterization at individual levels, thus yielding results with a potentially high level of clinical translation. Moreover, as inherently multivariate approaches, supervised ML methods are sensitive to spatially distributed and subtle effects in the brain, which would be undetectable if we were to use traditional univariate methods which focus on gross differences at the group level [22].

In the present study, we hypothesized that some common features of patients with alexithymia might be good predictors of alexithymia. Among these features, the distinct relationship of alexithymia with somatization, anxiety, and depression has also been a research priority [2, 5, 11, 28–31]. A significant relationship has been reported between somatization and alexithymia in clinical and nonclinical samples [2]. It has been shown that individuals with alexithymia have aggravated depressive symptoms [32]. Other studies [29, 30] also support the hypothesis that the presence of alexithymia predisposes individuals to depression. Based on some evidence, anxiety or depression can trigger a reactive regression to emotional development, thereby developing alexithymic features [31].

Given the association of alexithymia with somatization, anxiety, and depression, it seems that the presence of any of the foregoing disorders can be indicative of alexithymia. Accordingly, it is assumed that some items of BDI-II, BAI, and the somatization subscale of SCL-90-R questionnaires can be used to predict alexithymia. Besides, alexithymic features within the context of defective emotion recognition and regulation could be considered as a potential risk factor for medical and psychiatric conditions [33, 34].

Facial emotion recognition (FER), as a process of identifying facial expressions, is one of the most important elements in social communications and interactions [35]. Facial expressions can represent our emotional states and affect the generation and regulation of emotional states and behaviors in response to environmental signals [36]. FER is impaired in patients with high levels of alexithymia [37–43]. In addition, alexithymic patients show less activity in areas of the brain involved in extracting emotional components from facial expressions and FER (amygdala, insula, inferior frontal gyrus, striatum, and middle temporal gyrus) [27, 38, 43]. Accordingly, given the role of alexithymia in psychopathology and psychiatry, as well as the association of alexithymia with FER defects, the present study aimed to investigate the relationship between FER deficits, somatization, depression, and anxiety with alexithymia levels. The level of alexithymia (TAS-20) was predicted using the FER task dataset and the somatization subscale of SCL-90-R, BDI-II, and BAI questionnaires, which had been implemented in the ML methods of the artificial neural network (ANN) and support vector machine (SVM).

## 2. Materials and Methods

### 2.1. Participants

The statistical population of this study included students of the University of Tabriz in the academic year 2019–2020. In the first phase of the study, a total of 388 students, 174 males (44.8%) and 214 females (55.2%), were selected via cluster sampling (age range = 19–35, *M*_age_ = 23.27, SD_age_ = 3.93). After receiving approval from the Local Ethics Committee (Department of Psychology, University of Tabriz), the students were informed about the aim of the research, their informed consent was obtained, and they completed the questionnaires. The inclusion criteria were age range of 19–35 and willingness to participate in the study. The exclusion criteria were age above 35 years or below 19 years, diagnosis of any psychiatric disorders, use of medications that affect attention or cognition, diagnosis of any medical illness, addiction to alcohol or other recreational drugs, and unwillingness to participate in the study.

In the second phase of the study, 26 participants (11 males and 15 females) who met the inclusion criteria were included in the experimental study. According to the inclusion and exclusion criteria, 29 age-matched healthy controls (including 6 males and 23 females) were selected. Scores above 60 (TAS > 60) were considered as participants with alexithymia and scores below that threshold were healthy controls (TAS < 60) in the TAS-20 scale [44].  Table 1 provides a descriptive summary of the demographic data and questionnaires scores for all participants in the two groups (alexithymia and healthy control).

### 2.2. Behavioral Data

A total of 55 participants were included and divided into two groups (Alex and HC) based on the cut-off point on the TAS-20 scale. According to this scaling, participants with a score above 60 (TAS > 60) entered the alexithymia group and those scoring below 60 (TAS < 60) entered the healthy control group.  Table 1 summarizes the subjects' clinical and demographic characteristics and shows the results of the Mann-Whitney *U* test for each of the variables entered in the ML model. The results of the Chi-square test showed that the two groups were not significantly different in terms of gender distribution (*χ*^2^ = 3.000, *p* = 0.08).

### 2.3. Instruments

#### 2.3.1. Somatization Subscale of the Symptom Checklist-90-Revised (SCL-90-R)

The questionnaire was primarily developed by Derogatis et al. [45], revised based on clinical experience and psychometric analysis, and finalized in 1976. Respondents had to answer 90 questions on a 5-point Likert scale (with 0 meaning “not at all” and 4 meaning “extremely”). The somatization subscale of the SCL-90-R is a 12-item list of common somatic symptoms. It has good reliability in different studies [46]. Akhavan-Abiri and Shaeiri [47] reported that the reliability of Cronbach's alpha coefficient was 0.87 in the student sample. The current study also indicated the reliability for this subscale with Cronbach's alpha of 0.86.

#### 2.3.2. Toronto Alexithymia Scale (TAS-20)

The TAS is a 20-item self-report questionnaire with three dimensions, namely, difficulty identifying feelings (DIF), difficulty describing feelings (DDF), and externally oriented thinking (EOT) [48]. Items are scored based on a 5-point Likert scale from strongly disagree (1) to strongly agree (5). The international cut-off values are as follows: 20–50 = nonalexithymic subjects, 51–60 = borderline alexithymic subjects, and 61–100 = alexithymic subjects [44]. In our study, subjects with a total TAS score over 60 were considered as alexithymic, and those with a score under 60 were considered as nonalexithymic [33]. In the Iranian sample, the reliability of this questionnaire based on Cronbach's alpha was 0.79 for the whole TAS-20 scale and 0.75, 0.71, and 0.66 for DIF, DDF, and EOT, respectively. The total validities of the TAS-20 scale and DIF, DDF, and EOT dimensions in the Iranian clinical samples using test-retest were 0.77, 0.73, 0.69, and 0.65, respectively [49].

#### 2.3.3. Beck Anxiety Inventory (BAI)

Beck et al. [50] introduced the BAI which specifically measures the severity of clinical anxiety symptoms. The BAI is a 21-item questionnaire in which participants are to select from four options that indicate the severity of anxiety. The four options for each question are scored on a 4-point Likert scale ranging from 0 to 3. Its internal consistency coefficient was 0.92, and its validity by a one-week test-retest was 0.75 [50]. In an Iranian population, Kaviani and Mousavi [51] reported a validity coefficient of 0.72, a test-retest reliability coefficient of 0.83, and a Cronbach's alpha of 0.92.

#### 2.3.4. Beck Depression Inventory-Second Edition (BDI-II)

Similar to its first edition, the questionnaire [52] comprises 21 items, where the individuals select one of the four options for each item that indicates the severity of their depression symptoms. Each item is also scored from 0 to 3. The 21 items of the BDI-II are classified into three groups: affective, physical, and cognitive symptoms. Beck et al. [52] reported an internal consistency of 0.73 to 0.92 and an alpha coefficient of 0.86 for the patient group and 0.81 for the nonpatient group. Dobson and Mohammad Khani [53] obtained an alpha coefficient of 0.92 for the outpatients and 0.93 for the students, and a one-week test-retest coefficient of 0.93 was obtained in an Iranian sample.

#### 2.3.5. Facial Emotion Recognition (FER) Task

The computer-based task is utilized to assess the recognition ability of facial emotional expressions. It was designed through the use of Python software and videos extracted from the Amsterdam Dynamic Facial Expression Set (ADFES), and it was validated by Hawk et al. [54].

**Figure 1:**
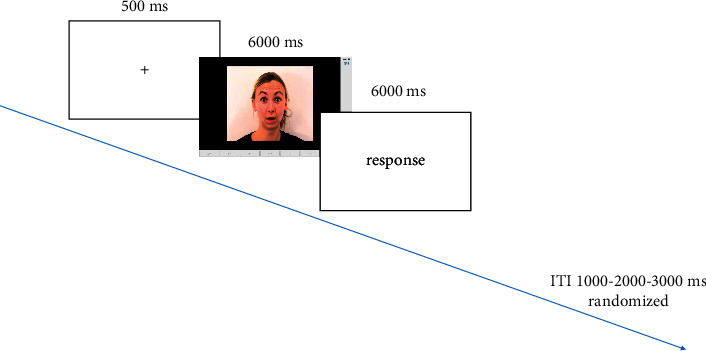
Facial emotion recognition (FER) task was used in this study. The total number of trials was 168. A fixation cross, appearing on the screen for 500 ms, was immediately followed by a dynamic facial expression presented for 6000 ms. The participants had to press the space bar as soon as they recognized the emotion, and they had 6 seconds to choose the type of emotion from the options.

The task starts with practice trials to allow the participants to become familiar with the main test. At the beginning of the experiment (Figure 1), a fixation cross was presented for 500 ms to locate the participants' visual attention. The practice section included 14 trials (videos of one male and one female presenter) and the main test included 168 trials (videos of 4 male and 4 female presenters with three random repetitions for each of the six basic emotions). Presenters with European and Asian faces presented each of the following expressions randomly: happiness, sadness, anger, disgust, fear, surprise, and no emotion. There was also a one-minute interval between the two stages of practice and the main test to eliminate the learning effect. In each section, videos were shown on a screen for 6 seconds. Participants were asked to press the space bar when they detected the emotional state first, and then they had 6 seconds to select the expressed emotion name, which was shown at the bottom of the screen.

### 2.4. Experimental Procedure

In the first phase of study, questionnaires were completed, and the participants were divided into the alexithymia group (called Alex) and healthy control group (called HC) based on the results of questionnaires. In the second phase, participants of both groups performed the FER task, which was conducted at the Emotion and Cognition Lab in the Department of Psychology, University of Tabriz. The characteristics of our sample (including the number of participants, mean, and standard deviation of our variable in each group) are presented in Table 1.

### 2.5. Classification Algorithms

The SVM and feedforward neural network (FNN) algorithms are two supervised learning classification methods that were used for classification in this study. SVM is a specific type of supervised ML method that aims to classify data points based on statistical learning theory through maximizing the margin between classes in a high-dimensional space [55–57]. An SVM classifier transforms the prediction problem into a square optimization problem, reducing the number of processes in the training phase and performing better and faster compared with other algorithms in terms of prediction accuracy [57, 58]. The FNN algorithm uses multiple features to predict a target variable via learning input data through their weights. FNNs are also inspired by the biological neural system with features, such as parallel computing, nonlinearity, adaptability, responsiveness, and fault tolerance [59]. An FNN is made up of a number of layers (one-layer or multilayer designs) and a number of neurons with behaviors similar to a biological neuron. SVM and FNN classifiers are opted for owing to their accurate performance on nonlinear and high-dimensional data and their wide usage in a variety of different machine learning applications, including psychiatry and psychology research [21, 23, 60].

### 2.6. Model Training and Performance Evaluation

Two sets of predictor variables were used to train each ML model: (1) all of the available data, namely, demographic information, SCL-90-R items, recognition, and time components of the FER task, and three subscales of depression and anxiety scores and (2) a subset of available data was selected based on the feature selection process, including SCL-5, neutral recognition, surprise recognition, fear time, disgust time, sadness time, anger time, depression physical symptoms, depression emotional symptoms, depression cognitive symptoms, and anxiety. The two sets of variables for training the ML models evaluate the necessity of all the predictor variables and determine the importance of the selected clinical scales during the feature selection process for classification between groups Alex and HC.

The k-fold cross-validation (*k* = 5, *k* = 10) technique was used to evaluate the generalizability of the models. This technique is typically used in ML approaches to compare and select a specific model for a given predictive modeling problem. In general, the estimations have a lower bias compared with other methods. Hyperparameter optimization was further adjusted using Bayesian optimization inside each cross-validation fold. This is an important procedure employed to classify new data as it allows the model to be simulated on unseen data. For SVM and FNN classifiers, the Kernel function and the number of layers and neurons are important hyperparameters, respectively; they are adjusted using k-fold cross validation. Model performance was assessed using the area under the curve (AUC), accuracy, sensitivity, specificity, and F1-measure.

To select the best features for accurate prediction, a sequential (backward) feature selection method was used. In the backward elimination, the model started with all features and the least significant features were removed at each iteration, which improved the performance of the model. The process stopped when no more performance was observed.

For the final evaluation of the ML models, 20% of the total sample was conserved as a test set through random selection. The remaining 80% was used to train the model in k-fold cross validation so as to optimize the model performance in every step. Afterwards, the final optimal classifiers were evaluated in the test set, which was not seen by the algorithm during the training procedure.

### 2.7. Confusion Matrix

**Figure 2:**
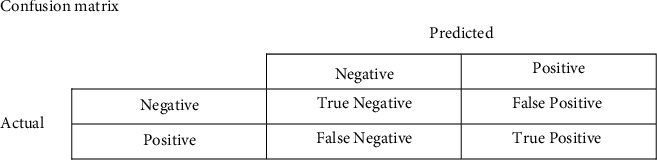
Confusion matrix. TN: True Negative, FP: False Positive, FN: False Negative, and TP: True Positive.

In order to represent the classification results, the confusion matrix was used. To compare the performance of the classification model, the following measurements were conducted: accuracy, precision, specificity, and recall (sensitivity) calculated on the basis of confusion matrix, as well as the F1-measure calculated based on the harmonic means of precision and recall. The following equations show the relationships between the confusion matrix and the performance measurements (Figure 2):

(1) $$\begin{array}{c} \text{Accuracy}=\;\frac{\text{TP}}{\text{TP}+\text{TN}+\text{FP}+\text{FN}},\\ \text{Precision}=\;\frac{\text{TP}}{\text{TP}+\text{FP}},\\ \text{Recal}\left(\text{sensitivity}\right)=\;\frac{\text{TP}}{\text{TP}+\text{FN}},\\ \text{specificity}=\;\frac{\text{TN}}{\text{TN}+\text{FP}},\\ F1\text{ measure}=\;\frac{2*\text{Recal}*\text{Precision}}{\text{Recal}+\text{Precision}} \end{array}$$

TP represents number of alexithymic patients detected correctly, TN represents number of healthy individuals detected correctly, FN represents number of alexithymic patients detected as healthy individuals, and FP represents number of healthy individuals detected as alexithymic patients.

### 2.8. Data Analysis

Data analysis was performed using SPSS and MATLAB v2017a. Of the 55 participants who were entered in the ML model, 29 individuals (6 males and 23 females with a mean age of 23.965 (SD = 5.697)) were placed in the HC group. Based on the cut-off point of the TAS-20 questionnaire, 26 individuals (11 males and 15 females with a mean age of 23.192 (SD = 5.557)) were assigned to the alexithymia group. Mann-Whitney *U* test was performed to compare the clinical variables between the two groups (Alex and HC); the effects sizes (*d*) were specified through Borenstein's formula [61]. Cohen's suggested benchmarks (small: *d* = 0.2, medium: *d* = 0.5, and large: *d* = 0.8) were also employed to interpret the magnitude of the effect sizes [62]. Furthermore, Chi-square test was utilized to compare categorical variables, such as gender.

**Figure 3:**
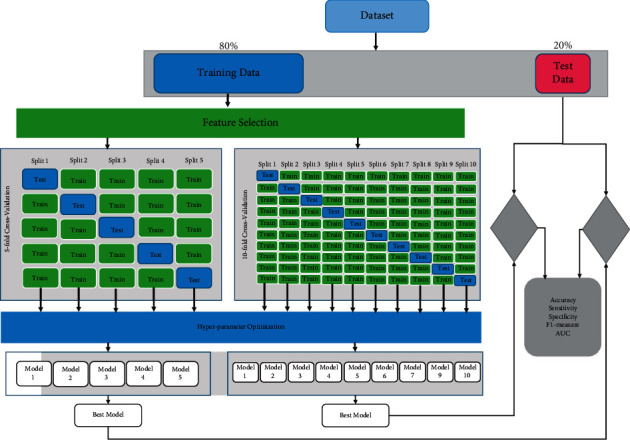
Machine learning procedure for training and testing the data.

As the first step of classification between the two groups, we ran classifiers including all the variables. In the second step, using the feature selection algorithm, the features increasing the classification accuracy were selected. The k-fold cross-validation technique was used to adjust the hyperparameters.  Figure 3 depicts the whole process of model training, evaluation, and testing in this study.

## 3. Results

### 3.1. Classification Models

Both the SVM and FNN classification algorithms were able to distinguish between Alex and HC. The confusion matrix for the classification models is shown in Table 2. The confusion matrix shown in Table 2 represents the values of performance for the final classification model using two different classifiers (FNN and SVM), two different evaluation methods (using 5-fold cross validation and 10-fold cross validation) without/with feature selection and hyperparameter tuning. In general, confusion matrices represent the finding of the distribution of all the predicted values and how to compare with their true values.

Table 3 shows the model performance for each classification algorithm using the 5-fold cross validation and 10-fold cross validation with and without feature selection and hyperparameter tuning. The SVM model had a significantly better performance with feature selection and hyperparameter tuning. This classifier, trained by 10-fold cross validation, performed better numerically, with a prediction accuracy of 81.8% and AUC of 0.80. The performance of the FNN model did not change significantly (prediction accuracy of 72.7% and AUC of 0.73) when trained with feature selection and hyperparameter tuning or without optimization.

Hyperparameter optimization for the SVM algorithm was performed using Bayesian optimization. Radial basis function (RBF) Kernel was selected to define the SVM model and the optimum hyperparameters for this model = 893.56 and Kernel scale = 6.03. Regarding the feedforward neural network, the settings included one hidden layer and 10 neurons. These hyperparameters are considered as the default for the simplest neural network.

Through the feature selection process, we also found the 11 most important predictors that would differentiate between the Alex and HC groups. These predictors were SCL-5, neutral recognition, surprise recognition, fear time, disgust time, sadness time, anger time, depression physical symptoms, depression emotional symptoms, depression cognitive symptoms, and anxiety. In order to specify the effect of optimization and feature selection on models, the classifiers were run with and without feature selection.

## 4. Discussion

The recent years have seen an increase in the use of new methods such as ML to diagnose a variety of medical diseases and psychiatric disorders; these approaches can replace tools, such as clinical judgment and questionnaires. In addition, the existing methods have some disadvantages that make it difficult to diagnose these conditions with more certainty. For instance, Mannarini et al. [20] showed the downsides of using TAS-20 and Toronto Structured Interview for Alexithymia (TSIA). Their findings indicated that TSIA was more time-consuming (minimum 40 min) and required training to be administered, and its accuracy was dependent on standardized administration and rating across different interviewers. The accuracy of the TAS-20 results also depends on the respondents' motivation and ability to reply sincerely [20]. Furthermore, the common statistical methods cannot diagnose disorders based on the existing data [21]. In contrast, ML models are based on feature selection to detect the optimum predictors of the disorders [22].

The objective of the present study was to use the most appropriate ML model capable of making the best distinction between alexithymic and healthy individuals. Previously, two studies predicted alexithymia utilizing machine learning models. Yöntem and Adem [63] applied an SVM model to predict the levels of alexithymia through automatic thoughts dataset. Their findings suggest that automatic thoughts would be helpful in the prediction of alexithymia. Orrù et al. [21] developed a machine learning model which increases the diagnosticity of alexithymia in patients with fibromyalgia. However, these studies predicted alexithymia through questionnaire scores and they did not utilize other neuropsychological measurements. In the present study, we implemented ML approach to predict alexithymia based on the FER task. Our findings support the hypothesis that utilizing ML techniques increases the ability to detect alexithymia through FER scores. As explained in the Results section, the traditional statistical analysis showed that most variables and items in this study had a significant range of effects on alexithymia.

On the other hand, classification models based on ML techniques had a higher accuracy in the range of 72.7–81.8% following feature selection and optimization. The classifier was able to (i) correctly classify the subjects into two groups (Alex and HC) and (ii) identify the most informative features and predictors. The findings of the current study suggested that the SVM model using 10-fold cross validation and feature selection yielded an accuracy of 81.8% (AUC = 0.8, *F*1 = 0.84). According to the literature, all of the most important predictors selected using the feature selection method are associated with one another; using a combination of these items in our measures will enable mental health professionals to predict alexithymia more accurately. Therefore, based on our findings, we are going to design a gadget that can help the assistants of psychologists or psychiatrists in clinical settings to (1) predict alexithymia without the need for prior training and (2) interpret the results without them being influenced by the administrator's bias.

As mentioned in the Introduction, alexithymia has relatively high prevalence in societies, is associated with a variety of psychological disorders, and reduces the effectiveness of treatment in a number of mental disorders. In addition, patients are likely to misinterpret their emotional arousal as a sign of disease [64]. This can lead to an overperception of the disease and patients seeking medical help, while there is no medical explanation for these symptoms [21]. Accordingly, improving the diagnostic methods of alexithymia, such as using ML models, can help therapists identify and present a professional, timely treatment and enhance the quality of life in these patients. The results might highlight the difficulty in identifying and detecting emotions, such as apathy and surprise and their reaction time in recognizing such emotions as fear, disgust, sadness, and anger. Among these, the recognition of neutral emotional states was notable. These findings could provide important implications for future research that should investigate the factors involved in the problems of alexithymic patients.

There were some limitations in this study. Our patients were not diagnosed according to a structured diagnostic interview; rather they were scored based on their self-report. The second limitation was the small size of our sample. Of the 388 participants who responded to the TAS, only 26 persons received a definite cut-off point for the diagnosis of alexithymia. Future replications with a larger sample size are necessary for more definitive conclusions. Besides, regarding the conflicting literature about gender differences in the prevalence of alexithymia, it is recommended that future studies should consider the role of gender in their results.

## 5. Conclusion

We used a combination of questionnaire items (somatization subscale from SCL-90-R, BDI-II, and BAI) and FER task to predict alexithymia, and these measurements complement each other and increase the prediction accuracy. Also, using our proposed gadget, the accuracy of the diagnosis (done by either the therapist or assistant therapist) increases and remains unaffected by factors such as assessor's bias and the difficulty in interpreting the result of interviews or questionnaires, and there is no need for prior training to interpret the findings of our gadget. Besides, one of the common problems in using questionnaires is that the subjects (patients) probably try to provide a positive image of themselves. In the FER task, however, this problem disappears due to the nature of the test that one only requires to choose the right emotion from the available options.

## Figures and Tables

**Table 1:** Descriptive statistics of demographic and questionnaire data for each group (Alex and HC), effect size (*d*), and results of Mann–Whitney *U* test.

	Alex (*N* = 26)	HC (*N* = 29)	Effect size (*d*)	Mann–Whitney *U* test	Sig.
Gender (male/female)	11/15	6/23	0.47	295.50	0.086
Age (M ± SD)	23.19 ± 5.55	23.965 ± 5.69	0.14	337.00	0.498
TAS-20 total score (M ± SD)	64.46 ± 3.88	34.689 ± 2.80	−0.62	.00	0.000
BDI-II-physical symp (M ± SD)	4.692 ± 3.31	1.413 ± 1.50	−1.25	142.00	0.000
BDI-II-emotional symp (M ± SD)	5.80 ± 4.13	1.827 ± 1.79	−1.22	148.00	0.000
BDI-II-cognitive symp (M ± SD)	6.07 ± 4.38	1.655 ± 2.19	−1.25	130.00	0.000
BAI (M ± SD)	12.96 ± 9.02	6.275 ± 5.59	−0.88	186.00	0.001
Happy recognition (M ± SD)	0.99 ± 0.00	0.997 ± 0.00	−0.06	353.50	0.379
Fear recognition (M ± SD)	0.74 ± 0.16	0.748 ± 0.17	0.001	368.00	0.879
Sadness recognition (M ± SD)	0.94 ± 0.03	0.939 ± 0.06	−0.05	347.50	0.607
Anger recognition (M ± SD)	0.89 ± 0.08	0.879 ± 0.13	−0.13	374.50	0.966
Neutral recognition (M ± SD)	0.98 ± 0.02	0.987 ± 0.02	−0.06	360.50	0.715
Disgust recognition (M ± SD)	0.82 ± 0.08	0.853 ± 0.11	0.33	283.50	0.111
Surprise recognition (M ± SD)	0.98 ± 0.02	0.982 ± 0.02	−0.12	341.50	0.454
Happy time (M ± SD)	2.26 ± 0.34	2.249 ± 0.56	−0.03	302.00	0.206
Fear time (M ± SD)	2.90 ± 0.57	2.794 ± 0.63	−0.19	293.00	0.157
Sadness time (M ± SD)	3.00 ± 0.54	2.941 ± 0.55	−0.11	337.00	0.500
Anger time (M ± SD)	2.93 ± 0.57	2.741 ± 0.53	−0.35	266.00	0.061
Neutral time (M ± SD)	3.54 ± 0.53	3.356 ± 0.63	−0.32	281.00	0.106
Disgust time (M ± SD)	2.70 ± 0.64	2.539 ± 0.65	−0.25	298.00	0.183
Surprise time (M ± SD)	2.32 ± 0.64	2.419 ± 0.62	0.14	362.00	0.800
SCL-Q1 (M ± SD)	1.15 ± 1.08	1.034 ± 1.17	−0.010	336.50	0.470
SCL-Q2 (M ± SD)	1.26 ± 1.18	0.862 ± 0.95	−0.37	306.50	0.209
SCL-Q3 (M ± SD)	0.653 ± 0.97	0.275 ± 0.75	−0.43	300.00	0.080
SCL-Q4 (M ± SD)	1.07 ± 1.09	0.655 ± 0.76	−0.44	301.50	0.172
SCL-Q5 (M ± SD)	0.57 ± 1.06	0.482 ± 0.94	−0.09	371.50	0.906
SCL-Q6 (M ± SD)	1.65 ± 1.35	0.689 ± 1.00	−0.80	215.00	0.004
SCL-Q7 (M ± SD)	0.23 ± 0.42	0.103 ± 0.30	−0.33	329.00	0.207
SCL-Q8 (M ± SD)	0.76 ± 1.06	0.586 ± 1.08	−0.17	324.50	0.308
SCL-Q9 (M ± SD)	0.92 ± 1.19	0.310 ± 0.54	−0.65	279.50	0.055
SCL-Q10 (M ± SD)	0.53 ± 1.06	0.413 ± 0.73	−0.13	373.00	0.933
SCL-Q11 (M ± SD)	1.30 ± 1.15	0.551 ± 1.02	−0.69	225.00	0.005
SCL-Q12 (M ± SD)	0.84 ± 1.12	0.137 ± 0.35	−0.83	253.50	0.008

*Note*. M: mean; SD: standard deviation; TAS-20 : Toronto alexithymia scale; BDI-II: Beck depression inventory; BAI: Beck anxiety inventory; SCL: somatization subscale of SCL-90-R.

**Table 2:** Confusion matrix of the final model by two different classifiers (FNN and SVM), two different evaluation methods (using 5-fold cross validation and 10-fold cross validation) without/with feature selection and hyperparameter tuning.

			Predicted			
			Without feature selection and optimization		With feature selection and optimization	
			Negative	Positive	Negative	Positive
Actual	10-Fold cross validation					
	SVM	Negative	4	1	3	1
		Positive	3	3	1	6
	FNN	Negative	2	2	3	3
		Positive	2	5	1	4
	5-Fold cross validation					
	SVM	Negative	4	0	3	1
		Positive	4	3	2	5
	FNN	Negative	2	3	2	3
		Positive	2	4	2	4

Note. TP represents number of alexithymic patients detected correctly, TN represents number of healthy individuals detected correctly, FN represents number of alexithymic patients detected as healthy individuals, and FP represents number of healthy individuals detected as alexithymic patients.

**Table 3:** Model measurements including accuracy, sensitivity, specificity, AUC, and F1-measure by two different classifiers (FNN and SVM), two different evaluation methods (using 5-fold cross validation and 10-fold cross validation) without/with feature selection and hyperparameter tuning.

	Without feature selection and optimization					With feature selection and optimization				
	Acc (%)	Sens	Spec	AUC	F1-measure	Acc (%)	Sens	Spec	AUC	F1-measure
*10-Fold cross validation*										
SVM	63.64	0.50	0.80	0.65	0.56	81.81	0.86	0.75	0.80	0.84
FNN	63.64	0.71	0.5	0.75	0.67	63.64	0.80	0.50	0.80	0.71

*5-Fold cross validation*										
SVM	63.64	0.43	1.00	0.71	0.51	72.72	0.71	0.75	0.73	0.72
FNN	54.00	0.67	0.40	0.43	0.60	54.54	0.67	0.40	0.53	0.60

*Note.* AUC stands for area under the curve in ROC analysis and F1-measure. FNN, feedforward neural network; SVM, support vector machine; Acc, accuracy; Sens, sensitivity; Spec, specificity.

## Data Availability

The raw data used in this study will be made available by the authors to other qualified researchers.
